# Efficacy and safety of IVIG in CIDP: Combined data of the PRIMA and PATH studies

**DOI:** 10.1111/jns.12302

**Published:** 2019-02-15

**Authors:** Ingemar S.J. Merkies, Ivo N. van Schaik, Jean‐Marc Léger, Vera Bril, Nan van Geloven, Hans‐Peter Hartung, Richard A. Lewis, Gen Sobue, John‐Philip Lawo, Billie L. Durn, David R. Cornblath, Jan L. De Bleecker, Claudia Sommer, Wim Robberecht, Mika Saarela, Jerzy Kamienowski, Zbigniew Stelmasiak, Björn Tackenberg, Orell Mielke, on Behalf of the PRIMA Trial Investigators and the PATH Study Group, A. Sabet, K. George, L. Roberts, R. Carne, S. Blum, R. Henderson, P. Van Damme, J. Demeestere, S. Larue, C. D'Amour, P. Kunc, M. Valis, J. Sussova, T. Kalous, R. Talab, M. Bednar, T. Toomsoo, I. Rubanovits, K. Gross‐Paju, U. Sorro, M. Saarela, M. Auranen, J. Pouget, S. Attarian, G. Le Masson, A. Wielanek‐Bachelet, C. Desnuelle, E. Delmont, P. Clavelou, D. Aufauvre, J. Schmidt, J. Zschuentzsch, C. Sommer, D. Kramer, O. Hoffmann, C. Goerlitz, J. Haas, M. Chatzopoulos, R. Yoon, R. Gold, P. Berlit, A. Jaspert‐Grehl, D. Liebetanz, A. Kutschenko, M. Stangel, C. Trebst, P. Baum, F. Bergh, J. Klehmet, A. Meisel, F. Klostermann, J. Oechtering, H. Lehmann, M. Schroeter, T. Hagenacker, D. Mueller, A. Sperfeld, F. Bethke, V. Drory, A. Algom, D. Yarnitsky, B. Murinson, A. Di Muzio, F. Ciccocioppo, S. Sorbi, S. Mata, A. Schenone, M. Grandis, G. Lauria, D. Cazzato, G. Antonini, S. Morino, D. Cocito, M. Zibetti, T. Yokota, T. Ohkubo, T. Kanda, M. Kawai, K. Kaida, H. Onoue, S. Kuwabara, M. Mori, M. Iijima, K. Ohyama, M. Baba, M. Tomiyama, K. Nishiyama, T. Akutsu, K. Yokoyama, K. Kanai, I.N. van Schaik, F. Eftimov, N.C. Notermans, N. Visser, C. Faber, J. Hoeijmakers, K. Rejdak, U. Chyrchel‐Paszkiewicz, C. Casanovas Pons, M. Antonia, J. Gamez, M. Salvado, C. Marquez Infante, S. Benitez, M. Lunn, J. Morrow, D. Gosal, T. Lavin, I. Melamed, A. Testori, S. Ajroud‐Driss, D. Menichella, E. Simpson, E. Chi‐Ho Lai, M. Dimachkie, R.J. Barohn, S. Beydoun, H. Johl, D. Lange, A. Shtilbans, S. Muley, S. Ladha, M. Freimer, J. Kissel, N. Latov, R. Chin, E. Ubogu, S. Mumfrey, T. Rao, P. MacDonald, K. Sharma, G. Gonzalez, J. Allen, D. Walk, L. Hobson‐Webb, K. Gable, J.L. De Bleecker, W. Robberecht, M. Saarela, J. Franques, J.‐M. Léger, R. Juntas Morales, C. Sommer, A. Nguento, J. Schmidt, Ch. Schrey, J. Kamienowski, Z. Stelmasiak, G. Zwolińska

**Affiliations:** ^1^ Department of Neurology Maastricht University Medical Center Maastricht The Netherlands; ^2^ Department of Neurology St Elisabeth Hospital Willemstad Curaçao; ^3^ Department of Neurology Amsterdam University Medical Centers, University of Amsterdam Amsterdam The Netherlands; ^4^ National Referral Center for Rare Neuromuscular Diseases Hôpital Pitié‐Salpêtrière and University Paris VI Paris France; ^5^ Ellen and Martin Prosserman Centre for Neuromuscular Diseases, Division of Neurology, Department of Medicine University Health Network, University of Toronto Toronto Canada; ^6^ Institute for Research and Medical Consultations Imam Abdulrahman Bin Faisal University Dammam Saudi Arabia; ^7^ Department of Medical Statistics and Bioinformatics Leiden University Medical Center Leiden The Netherlands; ^8^ Department of Neurology, UKD and Center for Neurology and Neuropsychiatry, LVR Klinikum, Medical Faculty Heinrich Heine University Düsseldorf Germany; ^9^ Department of Neurology Cedars‐Sinai Medical Center Los Angeles California; ^10^ Department of Neurology Nagoya University Graduate School of Medicine Nagoya Japan; ^11^ CSL Behring Marburg, Germany, and King of Prussia Pennsylvania; ^12^ Department of Neurology Johns Hopkins University School of Medicine Baltimore Maryland; ^13^ Department of Neurology, AZ St‐Lucas Ghent Belgium; ^14^ Department of Neurology Universitätsklinikum Würzburg Würzburg Germany; ^15^ Department of Neurosciences, UZ Leuven Leuven Belgium; ^16^ Department of Neurology Helsinki University Central Hospital Helsinki Finland; ^17^ Dolnośląski Szpital Specjalistyczny Wrocław Poland; ^18^ Department of Neurology, Samodzielny Publiczny Szpital Kliniczny Lublin Poland; ^19^ Department of Neurology Philipps University Marburg Germany; ^20^ Gold Coast Hospital and Health Service Southport QLD; ^21^ St Vincent's Hospital Melbourne VIC; ^22^ Royal Brisbane & Women's Hospital Herston QLD; ^23^ UZ Leuven‐Neurologie Leuven; ^24^ Hopital Charles LeMoyne, Recherche Sepmus QC; ^25^ Neurologicka klinika Fakultni nemocnice Hradec Kralove Hradec Kralove; ^26^ Neurologicka klinika Prague; ^27^ Privatni ordinace neurologie Hradec Kralove; ^28^ East Tallinn Central Hospital Tallinn; ^29^ West Tallinn Central Hospital Tallinn; ^30^ Helsinki University Central Hospital Helsinki; ^31^ Hôpital de la Timone Neurologi Marseille; ^32^ Hôpital Haut‐Lévéque Bordeaux; ^33^ Hôpital Archet 1 Centre de référence maladies neuromusculaires Nice; ^34^ Centre hospitalier universitaire Hôpital Gabriel Montpied Clermont‐Ferrand; ^35^ Universitätsmedizin Göttingen Göttingen; ^36^ Universitaetsklinikum Wurzburg Wurzburg; ^37^ St Josefs‐Krankenhaus Potsdam; ^38^ Jüdisches Krankenhaus Berlin Berlin; ^39^ Klinikum der Ruhr‐Universität Bochum Bochum; ^40^ Alfried Krupp Krankenhaus Rüttenscheid Essen; ^41^ Georg‐August‐Universitätsmedizin Göttingen Göttingen; ^42^ Medizinische Hochschule Hannover Hannover; ^43^ Universitaetsklinikum Leipzig Leipzig; ^44^ Klinik und Poliklinik für Neurologie Charité‐Universitätsmedizin Berlin Berlin; ^45^ Charite Universitaetsmedizin Berlin; ^46^ Universitätsklinikum Köln; ^47^ Universitätsklinikum Essen Essen; ^48^ Klinikum Ibbenbüren Ibbenbüren; ^49^ Tel Aviv Sourasky Medical Center Tel Aviv; ^50^ Rambam Health Care Campus Haifa; ^51^ Policlinico SS Annunziata Chieti Scalo; ^52^ Ospedaliero Universitaria Careggi Firenze; ^53^ Azienda Ospedaliera Universitaria San Martino di Genova Genoa; ^54^ Fondazione Istituto DiRicovero Milano; ^55^ Azienda Ospedaliera S Andrea Universita degli Studi di Roma “La Sapienza” Rome; ^56^ Azienda ospedaliero universitaria San Giovanni Battista Torino; ^57^ Tokyo Medical and Dental University Tokyo; ^58^ Yamaguchi University Hospital Yamaguchi; ^59^ National Defense Medical Hospital Saitama; ^60^ Chiba University Hospital Chiba; ^61^ Nagoya University Hospital Nagoya; ^62^ Aomori Prefectural Central Hospital Aomori; ^63^ Kitasato University Hospital Kanagawa; ^64^ Juntendo University Hospital Tokyo; ^65^ Amsterdam University Medical Centers University of Amsterdam Amsterdam; ^66^ University Medical Center Utrecht Utrecht; ^67^ Maastricht University Medical Center Maastricht; ^68^ Samodzielny Publiczny Szpital Kliniczny Lublin; ^69^ Universitari de Bellvitge Servicio de Neurología Barcelona; ^70^ Hospital Universitario Vall d'Hebron Servicio de Neurología Barcelona; ^71^ Hospital Universitario Virgen del Rocío Seville; ^72^ National Hospital for Neurology and Neurosurgery London; ^73^ Salford Royal Hospital Salford; ^74^ IMMUNOe International Research Centers Centennial CO; ^75^ Northwestern University Feinberg School of Medicine Chicago IL; ^76^ Methodist Neurological Institute Houston TX; ^77^ University of Kansas Medical Center Kansas City KS; ^78^ University of Southern California Keck School of Medicine Los Angeles CA; ^79^ Hospital for Special Surgery New York NY; ^80^ St Joseph's Hospital and Medical Center Phoenix AZ; ^81^ Wexner Medical Center at the Ohio State University Columbus OH; ^82^ Weill Medical College of Cornell University New York NY; ^83^ University of Alabama Medical Center Birmingham Birmingham AL; ^84^ The Neurologic Institute Charlotte NC; ^85^ University of Miami Miami FL; ^86^ Department of Neurology University of Minnesota Minneapolis MN; ^87^ Duke University Medical Center Durham NC; ^88^ AZ St‐Lucas Gent; ^89^ UZ Leuven Leuven; ^90^ HUS Meilahti Hospital Helsinki; ^91^ Hôpital de la Timone Neurologie et Maladies Neuro‐Musculaire Marseille; ^92^ Groupe Hospitalier Pitié‐Salpêtrière Unité de Pathologie Neuro‐Musculaire Paris; ^93^ CHRU Hôpital Gui de Chauliac Montpellier; ^94^ Universitätsklinikum Würzburg Würzburg; ^95^ ASKLEPIOS Klinikum Uckermark GmbH Schwedt; ^96^ Universtitätsmedizin Göttingen Georg‐August‐Universität Göttingen; ^97^ Facharzt für Neurologie Berlin; ^98^ Dolnóslàski Szpital Specjalistyczny Wrocław; ^99^ Samodzielny Publiczny Szpital Kliniczny Lublin; ^100^ Centrum Neurologii Klinicznej Kraków

**Keywords:** CIDP, efficacy, IVIG, PATH, PRIMA

## Abstract

Intravenous immunoglobulin (IVIG) is a potential therapy for chronic inflammatory demyelinating polyneuropathy (CIDP). To investigate the efficacy and safety of the IVIG IgPro10 (Privigen) for treatment of CIDP, results from Privigen Impact on Mobility and Autonomy (PRIMA), a prospective, open‐label, single‐arm study of IVIG in immunoglobulin (Ig)‐naïve or IVIG pre‐treated subjects (NCT01184846, n = 28) and Polyneuropathy And Treatment with Hizentra (PATH), a double‐blind, randomized study including an open‐label, single‐arm IVIG phase in IVIG pre‐treated subjects (NCT01545076, IVIG restabilization phase n = 207) were analyzed separately and together (n = 235). Efficacy assessments included change in adjusted inflammatory neuropathy cause and treatment (INCAT) score, grip strength and Medical Research Council (MRC) sum score. Adverse drug reactions (ADRs) and ADRs/infusion were recorded. Adjusted INCAT response rate was 60.7% in all PRIMA subjects at Week 25 (76.9% in IVIG pre‐treated subjects) and 72.9% in PATH. In the pooled cohort (n = 235), INCAT response rate was 71.5%; median time to INCAT improvement was 4.3 weeks. No clear demographic differences were noticed between early (responding before Week 7, n = 148) and late responders (n = 21). In the pooled cohort, median change from baseline to last observation was −1.0 (interquartile range −2.0; 0.0) point for INCAT score; +8.0 (0.0; 20.0) kPa for maximum grip strength; +3.0 (1.0; 7.0) points for MRC sum score**.** In the pooled cohort, 271 ADRs were reported in 105 subjects (44.7%), a rate of 0.144 ADRs per infusion. This analysis confirms the efficacy and safety of IgPro10, a recently FDA‐approved IVIG for CIDP, in a population of mainly pre‐treated subjects with CIDP [Correction added on 14 March 2019 after first online publication: the INCAT response rate has been corrected.].

## 1 INTRODUCTION

Chronic inflammatory demyelinating polyneuropathy (CIDP) is characterized by symmetrical, proximal, and distal weakness or somatosensory alterations in the arms and legs that worsens over time.^1^, ^2^ The annual incidence of CIDP is estimated to be between 0.5 and 1.6 cases per 100 000 individuals and peak prevalence is between 40 and 60 years of age, with rates ranging from 1.6 to 8.9 per 100 000 adults in different regions.^3^, ^4^, ^5^, ^6^, ^7^, ^8^ CIDP occurs more commonly in men than women.^1^

The goals of CIDP treatment are to reduce symptoms, improve functional ability, prevent relapse, and maintain long‐term remission. Immunoglobulins (Igs), corticosteroids, and plasma exchange are considered as first‐line therapies.^9^ Intravenous Ig (IVIG) was suggested to be efficacious vs plasma exchange, prednisolone, and placebo in several small trials, and in a Cochrane review, including five of these trials, totaling 235 subjects (mainly treatment‐naïve) provided evidence that more subjects had improvements in disability with IVIG treatment (44%) vs placebo (18%).^10^ Included in these five studies was the landmark immune globulin intravenous CIDP efficacy (ICE) trial, which reported efficacy in a randomized trial considered large for the CIDP disease area.^11^ This double‐blind study, performed in 117 CIDP subjects, reported a significantly higher adjusted inflammatory neuropathy cause and treatment (INCAT) improvement rate (54%) with IVIG compared with placebo (21%). Recently, a Japanese study (n = 49) also reported IVIG efficacy with long‐term treatment (induction IVIG 2 g/kg bodyweight [bw] over five consecutive days, maintenance IVIG 1.0 g/kg bw every 3 weeks for up to 52 weeks). The study reported a response rate of 78% at 28 weeks, with a 10.5% relapse rate within the population that continued treatment to Week 52.^12^

IgPro10 (Privigen; CSL Behring, King of Prussia, Pennsylvania), a 10% human IVIG, recently approved by the FDA for CIDP, was first shown to be effective for the treatment of CIDP in the Privigen Impact on Mobility and Autonomy (PRIMA) study.^13^ This single‐arm study, performed in 28 subjects, reported clinical responses to IgPro10 that were similar to those in the IVIG arm of the ICE trial. Further evidence of the efficacy of IgPro10 in CIDP was provided by the IVIG phase of the recent Polyneuropathy And Treatment with Hizentra (PATH) study.^14^ Subjects were restabilized on IgPro10 after determining individual Ig dependency, prior to randomization to subcutaneous Ig (SCIG) or placebo.

In the current analysis, results from the PRIMA and PATH studies are combined, aiming to determine whether the findings in the smaller PRIMA study would be validated by the much larger PATH trial, with the final objective to confirm the efficacy and safety of IgPro10 for the treatment in a much larger group of patients with CIDP. The value of this combined analysis is that it leverages similarities in study design between the two studies, for example, same dosing paradigm and endpoints, to analyze the efficacy of IVIG in a large, combined patient cohort.

## 2 METHODS AND MATERIALS

### 2.1 PRIMA

PRIMA was a prospective, open‐label, single‐arm study, with the purpose of obtaining marketing approval for IVIG in CIDP in Europe.^13^ The efficacy and safety of IgPro10, for both induction therapy and maintenance therapy, were investigated. A total of 28 subjects (IVIG pre‐treated, n = 13; previously untreated, n = 15) received one induction dose of IgPro10 (2.0 g/kg bw) over 2 to 5 consecutive days, and up to seven maintenance doses of IgPro10 (1.0 g/kg bw) given every 3 weeks on 1 day or 2 consecutive days (total treatment period, 21 weeks). Pre‐treated subjects required a 10‐week wash‐out period prior to enrollment.

### 2.2 PATH

The same dosing regimen from PRIMA was used in 207 IVIG pre‐treated subjects during the IVIG restabilization phase (10–13 weeks) of the PATH study.^14^ Study participants completed this phase before being randomized to maintenance therapy with SCIG or placebo. Subjects first completed a 4‐ to 12‐week IVIG‐dependency test period, receiving no IgG therapy (wash‐out period). Subjects showing a deterioration (increase in adjusted INCAT by ≥1 before amendment 3; after amendment 3: increase in adjusted INCAT by ≥1, decrease in inflammatory Rasch‐built overall disability scale (I‐RODS) by ≥4 points, or decrease in grip strength by ≥8 kPa) progressed to the subsequent IgPro10 restabilization period, in which subjects initially received an IgPro10 induction dose of 2 g/kg bw, administered over 2 to 5 consecutive days, with a maximum of 1 g/kg bw on a single day. This was followed by maintenance doses of 1 g/kg bw every 3 weeks, given on 1 day or over 2 consecutive days, during Weeks 4, 7, and 10. Depending on the time needed to achieve IgPro10 restabilization, a fourth dose could be given during Week 13.

### 2.3 Patients

The inclusion and exclusion criteria were generally similar for the two studies.^13^, ^14^ The main inclusion criteria were age >18 years and definite or probable CIDP according to the European Federation of Neurological Societies/Peripheral Nerve Society (EFNS/PNS) 2010 criteria.^9^ Subjects with polyneuropathy of other causes, diseases that may cause neurological symptoms, or a history of thrombotic episodes were excluded. An important difference between the studies was the enrollment of only pre‐treated subjects in PATH, compared with pre‐treated and treatment‐naïve subjects in PRIMA.

### 2.4 Outcome measures and response criteria

A ≥1 point decrease (indicating improvement) from reference visit value in adjusted INCAT disability score was considered to be a response criterion in both studies (response had to be observed at last observation in PRIMA to be classed as a responder; in PATH response was recorded if the patient improved at any visit during the 13‐week observation period). Changes in maximum grip strength (using a Vigorimeter from Martin; Tuttlingen, Germany) and Medical Research Council (MRC) sum score were also assessed.

Treatment response for grip strength was defined in the PATH study as an improvement of ≥8 kPa. No grip strength‐related responder criteria were prospectively defined in PRIMA, however improvement of ≥8 kPa was used in a post‐hoc analysis.^15^ For MRC sum score, improvement was defined as an increase of ≥3 points in both studies.

Treatment‐emergent adverse events (AEs) and adverse drug reactions (ADRs) were assessed in both studies, in terms of the number of events per infusion as well as percentages of subjects affected. AEs were defined as ADRs if they were temporally associated with study intervention (from start of infusion up to 72 hours after the end of infusion) or considered to be causally related to IgPro10.

### 2.5 Pooled analysis statistical methodology

No formal hypotheses were tested. Confidence intervals (CIs) were not adjusted for multiplicity and are provided for explorative purposes. Median time to first response was calculated using all subjects in a Kaplan‐Meier analysis; subjects without response were censored at the date of their last visit. A post‐hoc analysis investigated baseline characteristics of early responders (those responding by INCAT within 7 weeks) vs those who responded after Week 7 using descriptive statistics. Week 7 cut‐off was based on clinical relevance. All analyses were done using the statistical analysis system (SAS) software package (SAS Institute, Cary, North Carolina) version 9.2 or higher.

## 3 RESULTS

### 3.1 Populations

The populations in PRIMA and PATH were similar in demographic and baseline characteristics and primary disease characteristics (Table 1). The main differences between the studies were the size of the study populations and pre‐study use of IVIG (46.4% [n = 13] of subjects in the PRIMA trial were pre‐treated with IVIG, while all subjects in PATH were pre‐treated with IVIG). When data from PRIMA and PATH were pooled, a total of 233 subjects were treated with IgPro10. Two subjects contributed data to both studies, giving a total pooled population of 235.

**Table 1:** Baseline demographics and patient characteristics of PRIMA and PATH

Parameter	PRIMA N = 28	PATH N = 207	Total N = 235
Demographic characteristics			
Sex, n (%)			
Female	10 (35.7)	76 (36.7)	86 (36.6)
Male	18 (64.3)	131 (63.3)	149 (63.4)
Race, n (%)			
White	28 (100.0)	186 (89.9)	214 (91.1)
Asian	0	17 (8.2)	17 (7.2)
American Indian or Alaska Native	0	1 (0.5)	1 (0.4)
Other	0	3 (1.4)	3 (1.3)
Age (years)			
Mean (SD)	58.7 (14.34)	56.5 (12.76)	56.8 (12.95)
Median (range)	58.0 (22–79)	58.2 (25–83)	58.0 (22–83)
Primary disease characteristics			
Diagnosis of definite CIDP^a^, n (%)	23 (82.1)	185 (89.4)	208 (88.5)
Time since diagnosis of CIDP (years), median (range)	2.1 (0.1–13.9)	3.0 (0.1–33.5)	2.7 (0.1–33.5)
Prior IVIG treatment, n (%)			
Pre‐treated	13 (46.4)	207 (100.0)	220 (93.6)
Untreated^b^	15 (53.6)	0	15 (6.4)
Screening INCAT total score (points), mean (SD)	2.9 (1.18)	2.7 (1.67)	2.8 (1.62)

Abbreviations: CIDP, chronic inflammatory demyelinating polyneuropathy; INCAT, inflammatory neuropathy cause and treatment; PATH, Polyneuropathy And Treatment with Hizentra; PRIMA, Privigen Impact on Mobility and Autonomy.

a According to European Federation of Neurological Societies/Peripheral Nerve Society diagnostic criteria.

b Subjects with newly diagnosed CIDP (developing over ≥2 months) or subjects with an IVIG treatment interruption for ≥1 year with a progressive disease (deteriorating in the last 2 months) before enrollment.

### 3.2 Efficacy

The response rate, based on adjusted INCAT scores in the overall PRIMA population was 60.7% at Week 25 (95% CI: 42.4–76.4), 76.9% in PRIMA pre‐treated subjects, and 46.7% in PRIMA treatment‐naïve subjects. The response rate in PATH subjects at Week 13 was 72.9% (95% CI: 66.5–78.5) as shown in Table 2. In the pooled cohort (n = 235), INCAT response rate was 71.5% (95% CI: 65.9–77.3) [Correction added on 14 March 2019 after first online publication: the INCAT response rate has been corrected.].

**Table 2:** Response rate by INCAT and MRC sum score in PRIMA and PATH

Response rate (%)	PRIMA			PATH	Pooled	
	Pre‐treated n = 13	Treatment‐naïve n = 15	Overall N = 28	N = 207	Pre‐treated n = 220	Overall N = 235
INCAT	76.9	46.7	60.7	72.9	73.2	71.5
MRC sum score	76.9	86.7	82.1	56.5	57.7	59.6
Max grip strength (dominant hand)	46.2	46.7	46.4	59.9	59.1	58.3

Abbreviations: INCAT, inflammatory neuropathy cause and treatment; MRC, Medical Research Council; PATH, Polyneuropathy And Treatment with Hizentra; PRIMA, Privigen Impact on Mobility and Autonomy.

**Figure 1:**
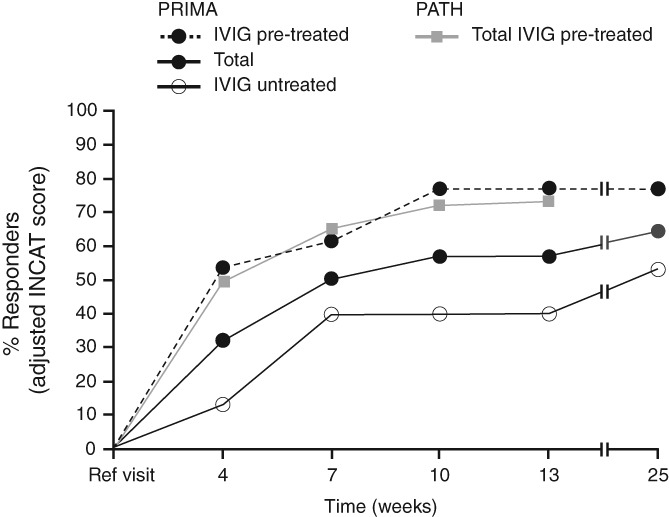
Cumulative INCAT response rate in PRIMA and PATH. INCAT, inflammatory neuropathy cause and treatment; IVIG, intravenous immunoglobulin; PATH, Polyneuropathy And Treatment with Hizentra; PRIMA, Privigen Impact on Mobility and Autonomy

The median time to first INCAT response was 3.0 weeks in PRIMA pre‐treated subjects, 18 weeks in PRIMA treatment‐naïve subjects and 3.7 weeks in PATH subjects. Cumulative INCAT response rate by time for the separate studies is presented in Figure 1 and INCAT response rate by week is shown in Table S1. In PRIMA IVIG pre‐treated subjects, 70.0% (7/10) of the responders responded after the induction dose (Week 4), and all pre‐treated responders responded by Week 10; 25% of the treatment‐naïve responders responded by Week 4, 75% by Week 7. In the overall population of PRIMA, 50.0% (9/18) of the responders at Week 25 responded after the induction dose (as assessed at Week 4), and all responders at Week 25 had responded by Week 19. In the PATH study, where all subjects had received IVIG pre‐treatment, 68.2% (103/151) of responders responded after the induction dose (Week 4), and all except two responders did so by Week 10. In the pooled cohort (n = 235), median time to first INCAT improvement was 4.3 weeks. A post‐hoc analysis was undertaken in the pooled analysis to evaluate subject baseline characteristics in early responders (those responding by INCAT by Week 7 [n = 148]) vs late responders (those responding after Week 7 [n = 21]). Early responders appeared to be slightly younger than late responders (55 years vs 61 years) and slightly more early responders were diagnosed with definite CIDP (93% vs 86%; Table S2).

In PRIMA, the median time to first MRC sum score improvement was 6 weeks in the overall population, 6 weeks in the treatment‐naïve population, and 3 weeks in the IVIG pre‐treated population. Improvement was seen in 82.1% of the overall population, 86.7% of the treatment‐naïve population, and 76.9% of the IVIG pre‐treated population. In PATH, the median time to first MRC improvement was 9.3 weeks; 56.5% of subjects improved in regards to their MRC score during the PATH IVIG restabilization phase. In the pooled cohort, median time to first MRC sum score improvement was also 9.3 weeks. In PRIMA, median time to first improvement in grip strength (dominant hand) was 7.1 weeks for pre‐treated subjects and 6.1 weeks for both the overall PRIMA population and treatment‐naïve population. In PATH, the median time to first improvement in grip strength was 9.3 weeks (seen in 54.9% of subjects). In the pooled cohort this was also 9.3 weeks.

Median improvements in outcome measures (baseline to last observation) for PRIMA pre‐treated subjects were: INCAT, −2.0 (25% and 75% percentile: −3.0; −1.0) points; grip strength, +5.0 (−9.0; 22.0) kPa; and MRC sum score, +5.0 (3.0; 10.0) points. For PRIMA treatment‐naïve subjects median changes were: INCAT, −1.0 (−2.0; 0.0) points; grip strength, +5.0 (−12.0, 33.0); and MRC sum score, +6.0 (3.0; 14.0). Corresponding results for PATH subjects were: INCAT, −1.0 (−2.0; 0.0) points; grip strength, +9.4 (1.3; 18.8) kPa; and MRC sum score, +3.0 (0.0; 6.0) points. In the pooled cohort (n = 235) median changes from baseline to last observation were: −1.0 (−2.0; 0.0) points for INCAT score; +8.0 (0.0; 20.0) kPa for grip strength; and +3.0 (1.0; 7.0) points for MRC sum score.

**Figure 2:**
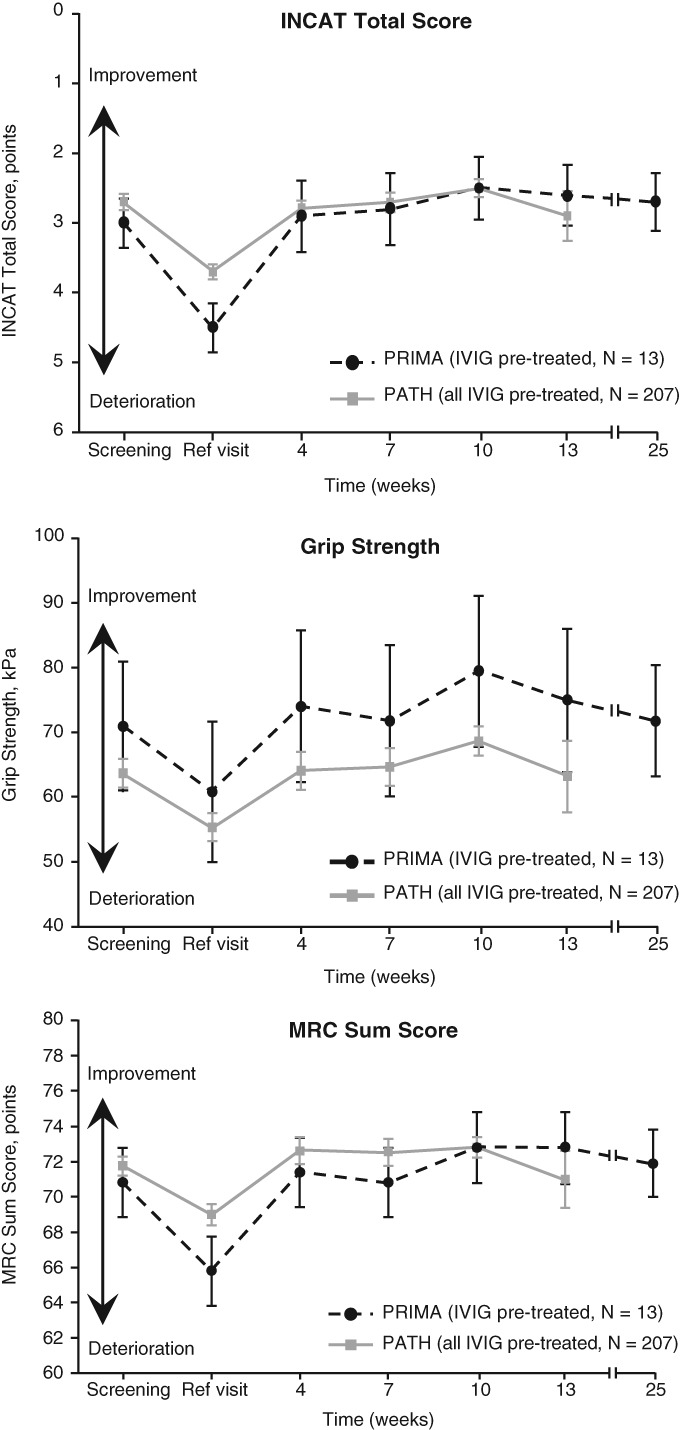
Mean change from baseline in INCAT total score, grip strength, and MRC sum score in PRIMA IVIG pre‐treated subjects and PATH. INCAT, inflammatory neuropathy cause and treatment; MRC, Medical Research Council; PATH, Polyneuropathy And Treatment with Hizentra; PRIMA, Privigen Impact on Mobility and Autonomy

Mean changes from baseline in INCAT total score, grip strength, and MRC sum score in PRIMA (IVIG pre‐treated subjects) and PATH are shown in Figure 2 **.**

### 3.3 Safety

Across the two studies, 1879 IgPro10 infusions were administered to 233 subjects: 259 infusions to 28 subjects in PRIMA and 1620 infusions to 207 subjects in PATH. In the PRIMA safety population (n = 28), 108 AEs occurred in 22 (78.6%) subjects (0.417/infusion). In IVIG pre‐treated PRIMA subjects (n = 13), 41 AEs occurred in 10 (76.9%) subjects (0.366/infusion), while 67 AEs were reported in 12 (80.0%) treatment‐naïve subjects (0.456/infusion). A total of 284 AEs in 100 (48.3%) subjects (0.175/infusion) were reported in the PATH safety population (n = 207). In both studies, the AE rate per infusion was lower during the maintenance treatment (0.387/infusion in PRIMA and 0.147/infusion in PATH) than during the induction treatment (0.493/infusion in PRIMA and 0.218/infusion in PATH).

Headache was the most frequent AE in both studies, seen in 9 (32.1%) PRIMA subjects (4 pre‐treated, 5 treatment‐naïve subjects) and 34 (16.4%) PATH subjects (overall 42/235 subjects [18.3%]). Causally related serious AEs were reported in 2 PRIMA subjects (hemolysis) and 7 PATH subjects (hypersensitivity, pulmonary embolism, increased blood pressure, exacerbation of CIDP, respiratory failure, rash, migraine).

In the pooled population, 271 ADRs were reported in 105 subjects (44.7%), representing a rate of 0.144 ADRs per infusion (Table 3). The most frequent ADRs were headache, nausea, hypertension and hemolysis. In PRIMA, 20 subjects (71.4%) had 71 ADRs; 85 subjects (41.1%) had 200 ADRs in PATH. There were no deaths during PRIMA or during the IgPro10 restabilization phase of PATH.

**Table 3:** Adverse drug reactions occurring in >5% of subjects in PRIMA (FAS) and PATH (PSDS)

Preferred term	PRIMA		PATH		Total	
	Number (%) of subjects N = 28	Number of events (rate per infusion) N = 259^a^	Number (%) of subjects N = 207	Number of events (rate per infusion) N = 1620^a^	Number (%) of subjects N = 235	Number of events (rate per infusion) N = 1879^a^
Any adverse drug reactions	20 (71.4)	71 (0.274)	85 (41.1)	200 (0.123)	105 (44.7)	271 (0.144)
Headache	8 (28.6)	19 (0.073)	32 (15.5)	50 (0.031)	40 (17.0)	69 (0.037)
Asthenia	4 (14.3)	4 (0.015)	2 (1.0)	2 (0.001)	6 (2.6)	6 (0.003)
Hypertension	4 (14.3)	6 (0.023)	5 (2.4)	6 (0.004)	9 (3.8)	12 (0.006)
Nausea	3 (10.7)	3 (0.012)	7 (3.4)	9 (0.006)	10 (4.3)	12 (0.006)
Pain in extremity	3 (10.7)	3 (0.012)	2 (1.0)	2 (0.001)	5 (2.1)	5 (0.003)
Hemolysis	2 (7.1)	2 (0.008)	7 (3.4)	7 (0.004)	9 (3.8)	9 (0.006)
Influenza‐like illness	2 (7.1)	2 (0.008)	0	0	2 (0.9)	2 (0.001)
Leukopenia	2 (7.1)	2 (0.008)	2 (1.0)	2 (0.001)	4 (1.7)	4 (0.002)
Rash	2 (7.1)	2 (0.008)	2 (1.0)	2 (0.001)	4 (1.7)	4 (0.002)

Abbreviations: FAS, full analysis set; N, number of subjects treated in the study or number of infusions; PATH, Polyneuropathy And Treatment with Hizentra; PRIMA, Privigen Impact on Mobility and Autonomy; PSDS, pre‐randomization safety data set.

Temporally associated events occurred during an infusion or within 72 hours after the end of infusion and were reported as “temporally related” in the source tables and listings of the PATH study.

a Number of infusions.

## 4 DISCUSSION

In the current analysis, combination of the data originating from the PRIMA and PATH studies resulted in the confirmation of the efficacy of IgPro10 based on changes seen in adjusted INCAT score, grip strength, and MRC sum score.^13^, ^14^ The findings in the PRIMA trial and findings from the larger cohort as part of the PATH trial simultaneously validated each other. Similar response rates and times to response with IgPro10 in IVIG pre‐treated subjects were seen in both studies, despite PATH having a shorter study period than PRIMA. In both studies, disability (measured by the INCAT score) and impairments (muscle strength as measured by grip strength and the MRC sum score) improved and remained stable after the IgPro10 induction dose or at subsequent visits.

At least half of subjects in both studies responded after just one induction dose of IVIG (Week 4). A total of 89% of the responders in PRIMA and 99% of the responders in PATH responded by Week 10 based on INCAT score (ie, after three infusions of IgPro10: one induction dose and two maintenance doses, given at a 3 week interval). Hence, the findings extend the knowledge that has been extracted from the ICE trial data suggesting most subjects may require several cycles (one induction and two maintenance doses) to respond to therapy.^16^ Latov et al showed among subjects classified as being responders that 47% had responded by Week 3 (corresponding to the induction dose), and the other 53% responded by Week 6 after a second infusion.^16^ The PRIMA–PATH pooled analysis shows that a substantial number of patients become responders after >6 weeks follow‐up; however, the data also show that response is highly unlikely after 10 weeks. This timing should be considered in clinical practice prior to deciding if a subject is not benefitting from treatment early in the treatment course. A recent study reported that 69% of subjects treated with IVIG for 52 weeks maintained INCAT improvement, further supporting the acute and long‐term use of IVIGs.^12^

This is the largest group of CIDP subjects that has undergone well‐defined outcome assessments. Previous Cochrane assessments of a similar number of subjects were mainly on treatment‐naïve subjects using a variety of outcome measures, whereas the current analysis comprised mainly pre‐treated subjects with the same outcome measures and the same IVIG induction and maintenance regimens. It should be considered that the previous ICE study included subjects untreated with IVIG for 3 months prior to the study as compared with the PRIMA population of which half were untreated for 1 year, and PATH in which all subjects were pre‐treated. The response rate in treatment‐naïve subjects in the PRIMA study (46.7%) is in a similar range to the 55% INCAT response rate reported in treatment‐naïve subjects in the Cochrane review.^10^ A higher response rate was observed in pre‐treated subjects in our combined analysis; the Kuwabara et al study, where all subjects were IVIG pre‐treated, had a responder rate of 77.6% at Week 28.^12^ It is also of note that in this combined analysis, there was a proportion of subjects who had previously responded to IVIG who did not respond well after the withdrawal (wash‐out) and re‐establishment of IVIG treatment. This highlights both the remitting–relapsing nature of CIDP and the importance of regular testing of Ig dependency in clinical practice to determine if a subject is still benefitting from IVIG treatment.

The safety data from both studies indicated that there were no unexpected AEs, almost all being categorized as mild and partially transient, in conformity with previous reports.^11^, ^17^ Hemolytic events were observed in both trials. The trials were undertaken before the implementation of an immunoaffinity chromatography step in the production process, which lowers isoagglutinin levels by 75% to 88%,^18^ and is expected to reduce the incidence of hemolytic events seen in future studies.^19^ The safety results from the combined analysis support that IgPro10 is a well‐tolerated treatment option when administered as induction and maintenance infusions to subjects with CIDP.

This analysis shows the efficacy and safety of IgPro10 in a combined data set of subjects with CIDP being treated with an IVIG. Our study confirms that the majority of those with CIDP who will respond to IVIG will do so after an induction dose plus at least 2 maintenance doses, and that a substantial number only respond after the second maintenance dose. Shorter regimens may not identify all who will respond to IVIG. Longer regimens “waiting” for a response may not be needed as late responders (after 10 weeks) are in fact unlikely.

## PATH STUDY GROUP

*Australia* A Sabet, K George (Gold Coast Hospital and Health Service, Southport, QLD). L Roberts, R Carne (St Vincent's Hospital, Melbourne, VIC). S Blum, R Henderson (Royal Brisbane & Women's Hospital, Herston, QLD). *Belgium* P Van Damme, J Demeestere (UZ Leuven‐Neurologie, Leuven). *Canada* S Larue, C D'Amour (Hopital Charles LeMoyne, Recherche Sepmus, Greenfield Park, QC). *Czech Republic* P Kunc, M Valis (Neurologicka klinika, Fakultni nemocnice Hradec Kralove, Hradec Kralove). J Sussova, T Kalous (Neurologicka klinika, Vseobecna fakultni nemocnice v Praze, Prague). R Talab, M Bednar (Privatni ordinace neurologie, Hradec Kralove). *Estonia* T Toomsoo, I Rubanovits (East Tallinn Central Hospital, Tallinn). K Gross‐Paju, U Sorro (West Tallinn Central Hospital, Tallinn). *Finland* M Saarela, M Auranen (Helsinki University Central Hospital, Helsinki). *France* J Pouget, S Attarian (Hôpital de la Timone Neurologi, Marseille). G Le Masson, A Wielanek‐Bachelet (Hôpital Haut‐Lévéque, Service de Neurologie Centre hospitalier universitaire de Bordeaux, Bordeaux). C Desnuelle, E Delmont (Hôpital Archet 1 Centre de référence maladies neuromusculaires, Nice). P Clavelou, D Aufauvre (Centre hospitalier universitaire Hôpital Gabriel Montpied, Clermont‐Ferrand). *Germany* J Schmidt, J Zschuentzsch (Universitätsmedizin Göttingen, Göttingen). C Sommer, D Kramer (Universitaetsklinikum Wurzburg, Wurzburg). O Hoffmann, C Goerlitz (St Josefs‐Krankenhaus, Potsdam). J Haas, M Chatzopoulos (Jüdisches Krankenhaus Berlin, Berlin). R Yoon, R Gold (Klinikum der Ruhr‐Universität Bochum, Bochum). P Berlit, A Jaspert‐Grehl (Alfried Krupp Krankenhaus Rüttenscheid, Essen). D Liebetanz, A Kutschenko (Georg‐August‐Universitätsmedizin Göttingen, Göttingen). M Stangel, C Trebst (Medizinische Hochschule Hannover, Hannover). P Baum, F Bergh (Universitaetsklinikum Leipzig, Leipzig). J Klehmet, A Meisel (Klinik und Poliklinik für Neurologie Charité‐Universitätsmedizin Berlin, Berlin). F Klostermann, J Oechtering (Charite Universitaetsmedizin Berlin). H Lehmann, M Schroeter (Universitätsklinikum, Köln). T Hagenacker, D Mueller (Universitätsklinikum Essen, Essen). A Sperfeld, F Bethke (Klinikum Ibbenbüren, Ibbenbüren). *Israel* V Drory, A Algom (Tel Aviv Sourasky Medical Center, Tel Aviv). D Yarnitsky, B Murinson (Rambam Health Care Campus, Haifa). *Italy* A Di Muzio, F Ciccocioppo (Policlinico SS Annunziata, Chieti Scalo). S Sorbi, S Mata (Ospedaliero Universitaria Careggi, Firenze). A Schenone, M Grandis (Azienda Ospedaliera Universitaria San Martino di Genova, Genoa). G Lauria, D Cazzato (Fondazione Istituto DiRicovero, Milano). G Antonini, S Morino (Azienda Ospedaliera S Andrea Universita degli Studi di Roma “La Sapienza”, Rome). D Cocito, M Zibetti (Azienda ospedaliero universitaria San Giovanni Battista, Torino). *Japan* T Yokota, T Ohkubo (Tokyo Medical and Dental University, Tokyo). T Kanda, M Kawai (Yamaguchi University Hospital, Yamaguchi). K Kaida, H Onoue (National Defense Medical Hospital, Saitama). S Kuwabara, M Mori (Chiba University Hospital, Chiba). M Iijima, K Ohyama (Nagoya University Hospital, Nagoya). M Baba, M Tomiyama (Aomori Prefectural Central Hospital, Aomori). K Nishiyama, T Akutsu (Kitasato University Hospital, Kanagawa). K Yokoyama, K Kanai (Juntendo University Hospital, Tokyo). *Netherlands* I N van Schaik, F Eftimov (Amsterdam University Medical Centers, University of Amsterdam, Amsterdam). N C Notermans, N Visser (University Medical Center Utrecht, Utrecht). C Faber, J Hoeijmakers (Maastricht University Medical Center, Maastricht). *Poland* K Rejdak, U Chyrchel‐Paszkiewicz (Samodzielny Publiczny Szpital Kliniczny, Lublin). *Spain* C Casanovas Pons, M Antonia (Universitari de Bellvitge Servicio de Neurología, Barcelona). J Gamez, M Salvado (Hospital Universitario Vall d'Hebron Servicio de Neurología, Barcelona). C Marquez Infante, S Benitez (Hospital Universitario Virgen del Rocío, Seville). *United Kingdom* M Lunn, J Morrow (National Hospital for Neurology and Neurosurgery, London). D Gosal, T Lavin (Salford Royal Hospital, Salford). *United States* I Melamed, A Testori (IMMUNOe International Research Centers, Centennial, CO). S Ajroud‐Driss, D Menichella (Northwestern University Feinberg School of Medicine, Chicago, IL). E Simpson, E Chi‐Ho Lai (Methodist Neurological Institute, Houston, TX). M Dimachkie, R J Barohn (University of Kansas Medical Center, Kansas City, KS). S Beydoun, H Johl (University of Southern California Keck School of Medicine, Los Angeles, CA). D Lange, A Shtilbans (Hospital for Special Surgery, New York, NY). S Muley, S Ladha (St Joseph's Hospital and Medical Center, Phoenix, AZ). M Freimer, J Kissel (Wexner Medical Center at the Ohio State University, Columbus, OH). N Latov, R Chin (Weill Medical College of Cornell University, New York, NY). E Ubogu, S Mumfrey (University of Alabama Medical Center Birmingham, Birmingham, AL). T Rao, P MacDonald (The Neurologic Institute, Charlotte, NC). K Sharma, G Gonzalez (University of Miami, Miami, FL). J Allen, D Walk (Department of Neurology, University of Minnesota, Minneapolis, MN). L Hobson‐Webb, K Gable (Duke University Medical Center, Durham, NC).

## PRIMA STUDY GROUP

*Belgium* J. L. De Bleecker, AZ St‐Lucas, Gent (5 patients); W. Robberecht, UZ Leuven, Leuven (3 patients). *Finland* M. Saarela, HUS Meilahti Hospital, Helsinki (3 patients). *France* J. Franques, Hôpital de la Timone, Neurologie et Maladies Neuro‐Musculaire, Marseille (2 patients); J.‐M. Léger, Groupe Hospitalier Pitié‐Salpêtrière Unité de Pathologie Neuro‐Musculaire, Paris (1 patient); R. Juntas Morales, CHRU Hôpital Gui de Chauliac, Montpellier (1 patient). *Germany* C. Sommer, Universitätsklinikum Würzburg, Würzburg (4 patients); A. Nguento, ASKLEPIOS Klinikum Uckermark GmbH, Schwedt (2 patients); J. Schmidt, Universtitätsmedizin Göttingen, Georg‐August‐Universität, Göttingen (1 patient); Ch. Schrey, Facharzt für Neurologie, Berlin (1 patient). *Poland* J. Kamienowski, Dolnóslàski Szpital Specjalistyczny, Wrocław (3 patients); Z. Stelmasiak, Samodzielny Publiczny Szpital Kliniczny, Lublin (3 patients); G. Zwolińska, Centrum Neurologii Klinicznej, Kraków (2 patients).

## Supporting information

Supporting Information [Correction added on 14 March 2019 after first online publication: the Supporting Information has been updated.].
