# Supplementary material for: Efficacy and safety of IVIG in CIDP: Combined data of the PRIMA and PATH studies
Source: J Peripher Nerv Syst. 2019 Feb 15;24(1):48–55. doi: 10.1111/jns.12302 (PMC6594229; doi:10.1111/jns.12302)
Supplement: Supplementary file 1 — Supporting Information [Correction added on 14 March 2019 after first online publication: the Supporting Information has been updated.]. [file JNS-24-48-s001.docx]

**Supplementary Materials**

Table S1. INCAT response by week in PRIMA and PATH and pooled cohorts

| **Week** | **PRIMA** | | | **PATH** | **Pooled** | |
| --- | --- | --- | --- | --- | --- | --- |
|  | **Pre-treated (N = 13)** | **Treatment-naïve (N = 15)** | **Overall (N = 28)** | **(N = 207)** | **Pre-treated (N = 220)** | **Overall**  **(N = 235)** |
| **1** | 0 (0.0%) | 0 (0.0%) | 0 (0.0%) | n/a | n/a | 0 (0.0%) |
| **4** | 7 (53.8%) | 2 (13.3%) | 9 (32.1%) | 103 (49.8%) | 108 (49.1%) | 110 (46.8%) |
| **7** | 8 (61.5%) | 6 (40.0%) | 14 (50.0%) | 135 (65.2%) | 143 (65.0%) | 149 (63.4%) |
| **10** | 10 (76.9%) | 6 (40.0%) | 16 (57.1%) | 149 (72.0%) | 159 (72.3%) | 165 (70.2%) |
| **≥13** | 10 (76.9%) | 8 (53.3%) | 18 (64.3%) | 151 (72.9%) | 161 (73.2%) | 169 (71.9%) |

Abbreviations: INCAT, inflammatory neuropathy cause and treatment; n/a, not applicable; n, number of subjects; PATH, Polyneuropathy And Treatment With Hizentra; PRIMA, Privigen Impact on Mobility and Autonomy.

**Table S2. Characteristics in early versus late responders in the pooled PRIMA and PATH cohort**

|  | **Early responders (n = 148)** | **Late responders (n = 21)** |
| --- | --- | --- |
| Sex male, n (%) | 94  (63.5) | 13  (61.9) |
| Race white, n (%) | 140  (94.6) | 20  (95.2) |
| Age mean, years (SD) | 55.15 (12.783) | 61.04 (10.543) |
| Definite CIDP, n (%) | 137  (92.6) | 18  (85.7) |
| Mean time since diagnosis, years (SD) | 4.89 (5.447) | 4.69 (5.240) |
| IVIG pre-treated, n (%) | 142  (95.9) | 19  (90.5) |
| Baseline INCAT score, mean score (SD) | 2.7 (1.62) | 3.0 (1.53) |

Abbreviations: CIDP, chronic inflammatory demyelinating polyneuropathy; INCAT, inflammatory neuropathy cause and treatment; IVIG, intravenous immunoglobulin; n, number of subjects; PATH, Polyneuropathy And Treatment With Hizentra; PRIMA, Privigen Impact on Mobility and Autonomy; SD, standard deviation.
